# Supplementary material for: The structure of the teleost Immunoglobulin M core provides insights on polymeric antibody evolution, assembly, and function
Source: Nat Commun. 2023 Nov 21;14:7583. doi: 10.1038/s41467-023-43240-z (PMC10663602; doi:10.1038/s41467-023-43240-z)
Supplement: Supplementary file 1 — Supplementary Information [file 41467_2023_43240_MOESM1_ESM.pdf]

Supplementary Information

The structure of the teleost Immunoglobulin M core provides insights on polymeric antibody evolution, assembly, and function

Mengfan Lyu[1], Andrey G. Malyutin[2,3,6], and Beth M. Stadtmueller[1,4,5] *

[1]Department of Biochemistry, University of Illinois Urbana-Champaign, Urbana, Illinois 61801 USA

[2]Division of Biology and Biological Engineering, California Institute of Technology, Pasadena, CA 91125 USA

[3]Beckman Institute, California Institute of Technology, Pasadena, CA 91125 USA

[4]Department of Biomedical and Translational Sciences, Carle Illinois College of Medicine, University of Illinois Urbana-Champaign, Urbana, Illinois 61801 USA

[5]Carl R. Woese Institute for Genomic Biology, University of Illinois, Urbana, Illinois, 61801 USA

[6]Present address, Takeda Pharmaceuticals, Cambridge, MA 02139 USA

*Address correspondence to Beth M. Stadtmueller bethms@illinois.edu

This PDF contains:

Supplementary Figures 1-6

Supplementary Tables 1-5

Uncropped gel image used in Supplementary Figures

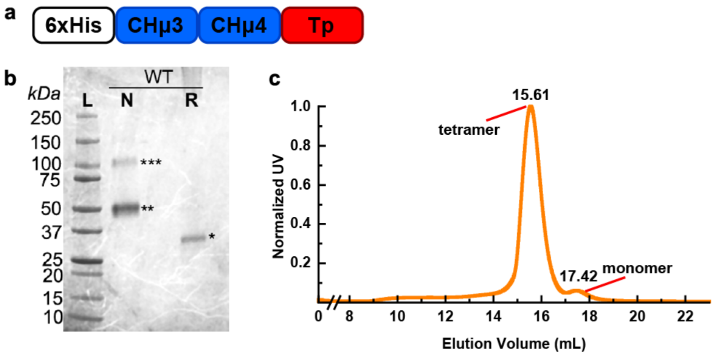

Supplementary Fig. 1 | Design of tFcμ construct and characterization of recombinant tFcμ expression. a Schematic of the tIgM heavy chain domains included in the tFcμ expression construct, including an N-terminal hexa-histidine tag. **b** SDS-PAGE gel analysis of wildtype tFcμ tetramer. *WT*, wildtype recombinant tFcμ protein. *L*, molecular weight standard. *N*, non-reducing conditions. *R*, reducing conditions. Putatively, bands correspond to *, single heavy chain. **, two disulfide-linked heavy chains. ***, higher order disulfide-linked heavy chains. **c** Size exclusion column chromatogram of recombinant tFcμ protein. Source data associated with panel **b** are provided in the Source Data file and the original gel image can be found at the end of the Supplementary Information file. Protein preparation, SDS-PAGE and SEC analysis has been repeated at least twice.

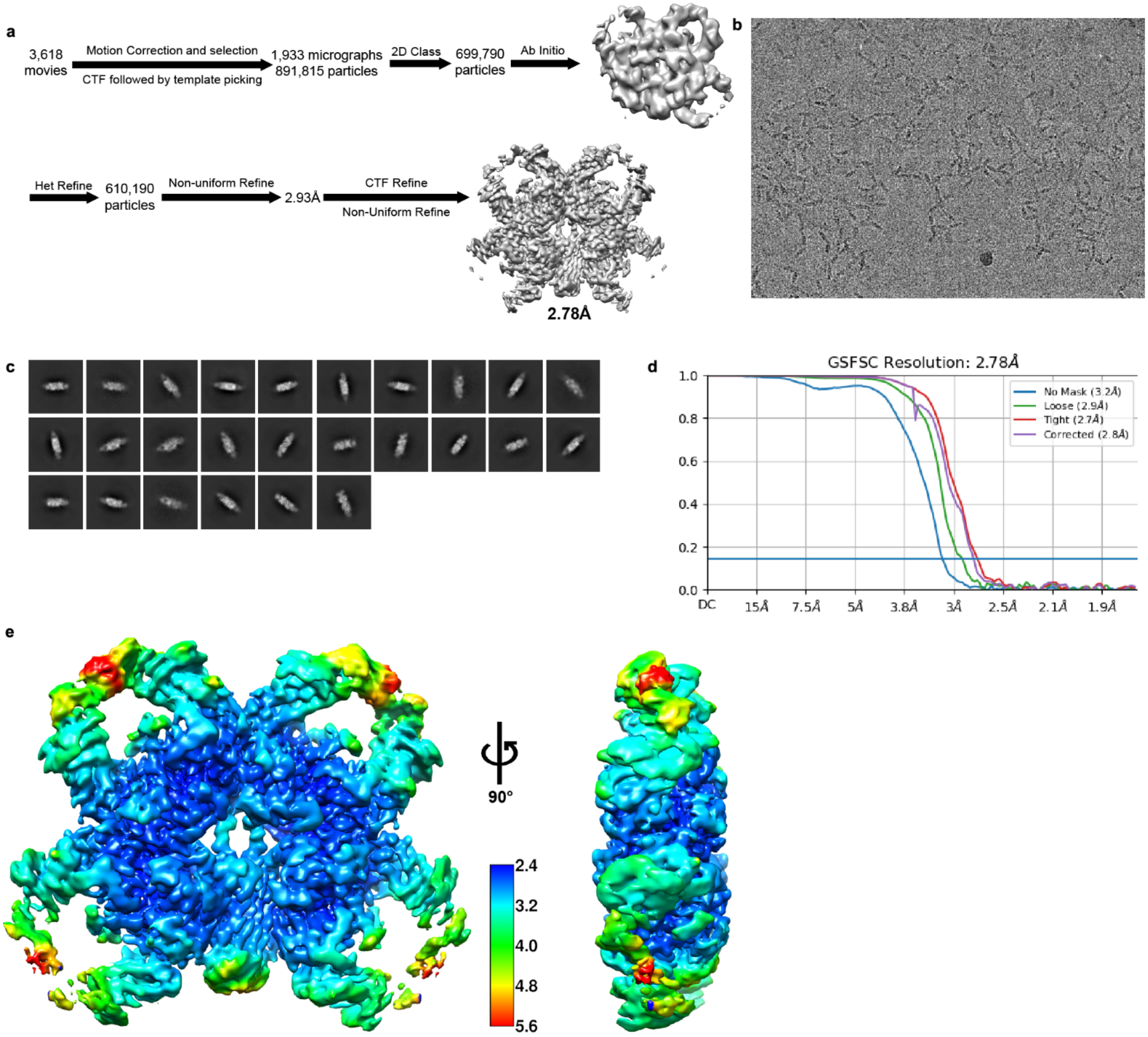

Supplementary Fig. 2 | tFcμ cryo-EM data collection, processing, and validation. a Schematic summary of tFcμ cryo-EM data processing pipeline in CryoSparc. **b** Representative micrograph after motion correction and selection. **c** Representative 2D class averages. **d** FSC curves for the final reconstruction with reported resolution at FSC = 0.143 shown by the blue horizontal line. **e** Local resolution map of the final reconstruction calculated in CryoSparc and rendered in UCSF Chimera; the unit of scale bar is given in Angstroms (Å).

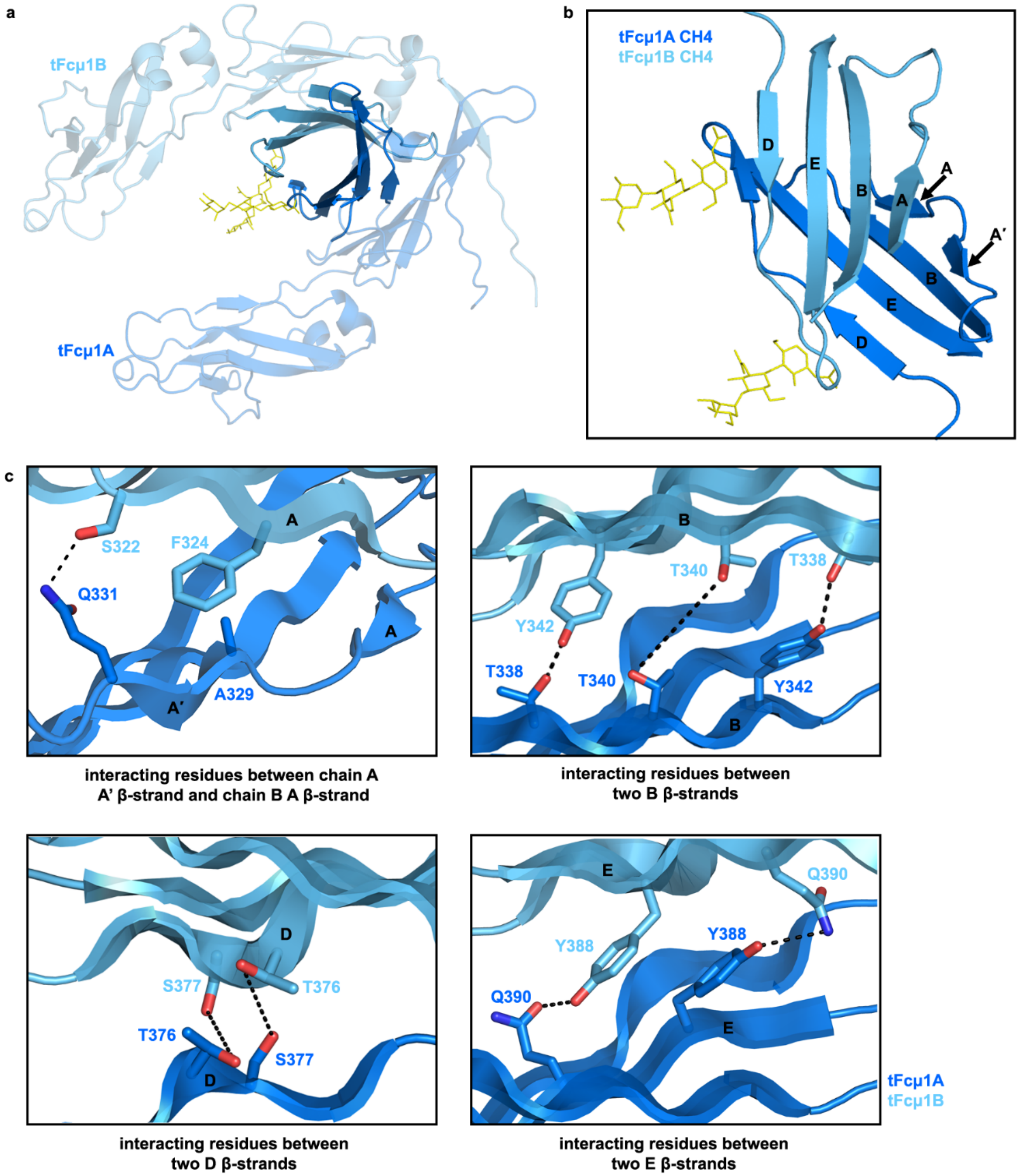

Supplementary Fig. 3 | Interface between two CHµ4 domains within Fcµ monomer. **a** Cartoon representation of tFcµ1 with glycans shown as yellow sticks. The intra-Fc tCHµ4-tCHµ4 interface is rendered using solid colors whereas the rest of the structure is rendered using transparent color. The orientation is the same as Fig. 1b. **b** Closeup view of intra-Fc tCHµ4-tCHµ4 interface between chain A and chain B with Ig-fold strand nomenclature labeled. Chain A contains A and A' strands whereas chain B contains only an A strand followed by a loop. **c** Interactions at intra-Fc tCHµ4-tCHµ4 interface between chain A and chain B. From left to right: interacting residues between two B, β-strands, two D, β-strands, and two E, β-strands; putative hydrogen bonds are shown as black, dashed lines.

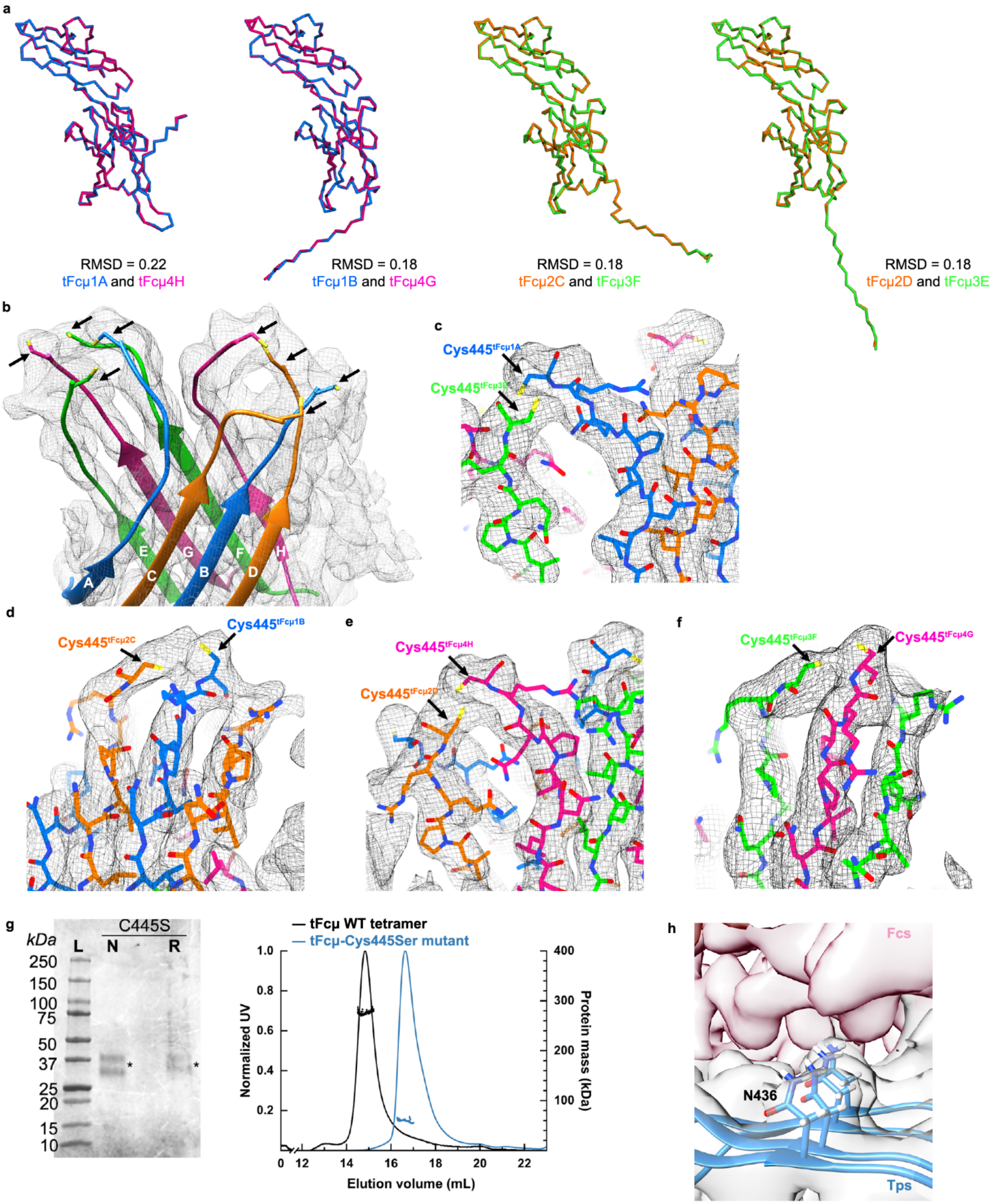

a
RMSD = 0.22
tFcμ1A and tFcμ4H
RMSD = 0.18
tFcμ1B and tFcμ4G
RMSD = 0.18
tFcμ2C and tFcμ3F
RMSD = 0.18
tFcμ2D and tFcμ3E
b
A B C D E F G H
c
Cys445tFcμ1A
Cys445tFcμ3E
d
Cys445tFcμ2C
Cys445tFcμ1B
e
Cys445tFcμ4H
Cys445tFcμ2D
f
Cys445tFcμ3F
Cys445tFcμ4G
g
kDa
C445S
L N R
250 150 100 75 50 37 25 20 15 10
tFcμ WT tetramer
tFcμ-Cys445Ser mutant
Normalized UV
Protein mass (kDa)
Elution volume (mL)
h
Fcs
N436
Tps

Supplementary Fig.4 | Local cryo-EM density of areas of interest and structural alignment of tFcμ chains. **a** Structural alignments between tFcμ chain A and chain H, chain B and chain G, chain C and chain F, chain D and chain E. **b-f** Cryo-EM density near tFcμ C-termini. The coloring scheme is the same as Fig. 1b. **b** Overview of C-termini density with side chains of Cys445 shown as sticks. **c-f** Density around potential interchain disulfide between $\text{Cys445}^{\text{Fcμ1A}}$ and $\text{Cys445}^{\text{Fcμ3E}}$, between $\text{Cys445}^{\text{Fcμ1B}}$ and $\text{Cys445}^{\text{Fcμ2C}}$, between $\text{Cys445}^{\text{Fcμ2D}}$ and $\text{Cys445}^{\text{Fcμ4H}}$, and between $\text{Cys445}^{\text{Fcμ3F}}$ and $\text{Cys445}^{\text{Fcμ4G}}$, respectively. **g** Characterization of tFcμ-Cys445Ser monomer. *Left*, SDS-PAGE gel. *WT*, wildtype recombinant tFcμ protein. C445S, tFcμ with Cys445Ser mutation. *L*, molecular weight standard. *N*, non-reducing condition. *R*, reducing condition. *, single heavy chain. The doublet band is attributed to heterogeneous glycosylation. *Right*, SEC-MALS chromatograms for wildtype tFcμ tetramer and tFcμ-Cys445Ser monomer. SEC elution profiles are shown as solid curves (left axis; normalized UV signal) and light scattering data, indicating protein mass, is shown as horizontal dots (right axis; protein mass in kDa). **h** Density of tFcμ Tp assembly around residue Asn436. Cartoon representation of $\text{Tp}^{\text{tFcμ1A}}$, $\text{Tp}^{\text{tFcμ1B}}$, $\text{Tp}^{\text{tFcμ2C}}$, and $\text{Tp}^{\text{tFcμ2D}}$ are shown. Density associated with non-Tp residues is shaded red. Source data associated with panel **g** are provided in the Source Data file and the original gel image can be found at the end of the Supplementary Information file. Protein preparation, SDS-PAGE and SEC analysis has been repeated at least twice.

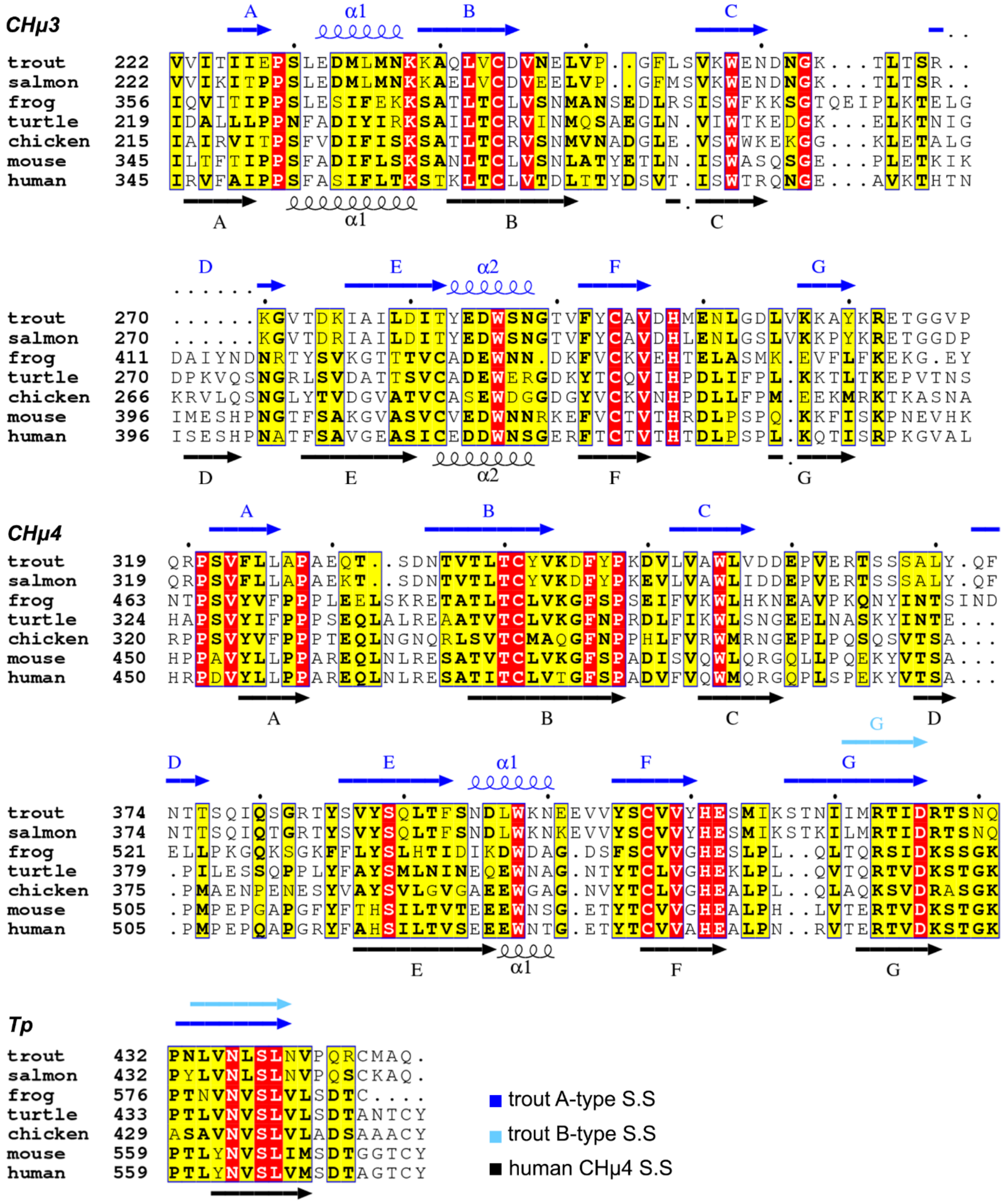

Supplementary Fig. 5 | Sequence alignment of CHμ3, CHμ4, and Tp residues. Sequence alignment of CHμ3, CHμ4 and Tp residues from trout, salmon, frog, turtle, chicken mouse, and human. Secondary structure (S.S) information for CHμ3, CHμ4, and the Tp are annotated. For the B-type tCHμ4 register, elements identical to A-type are not included.

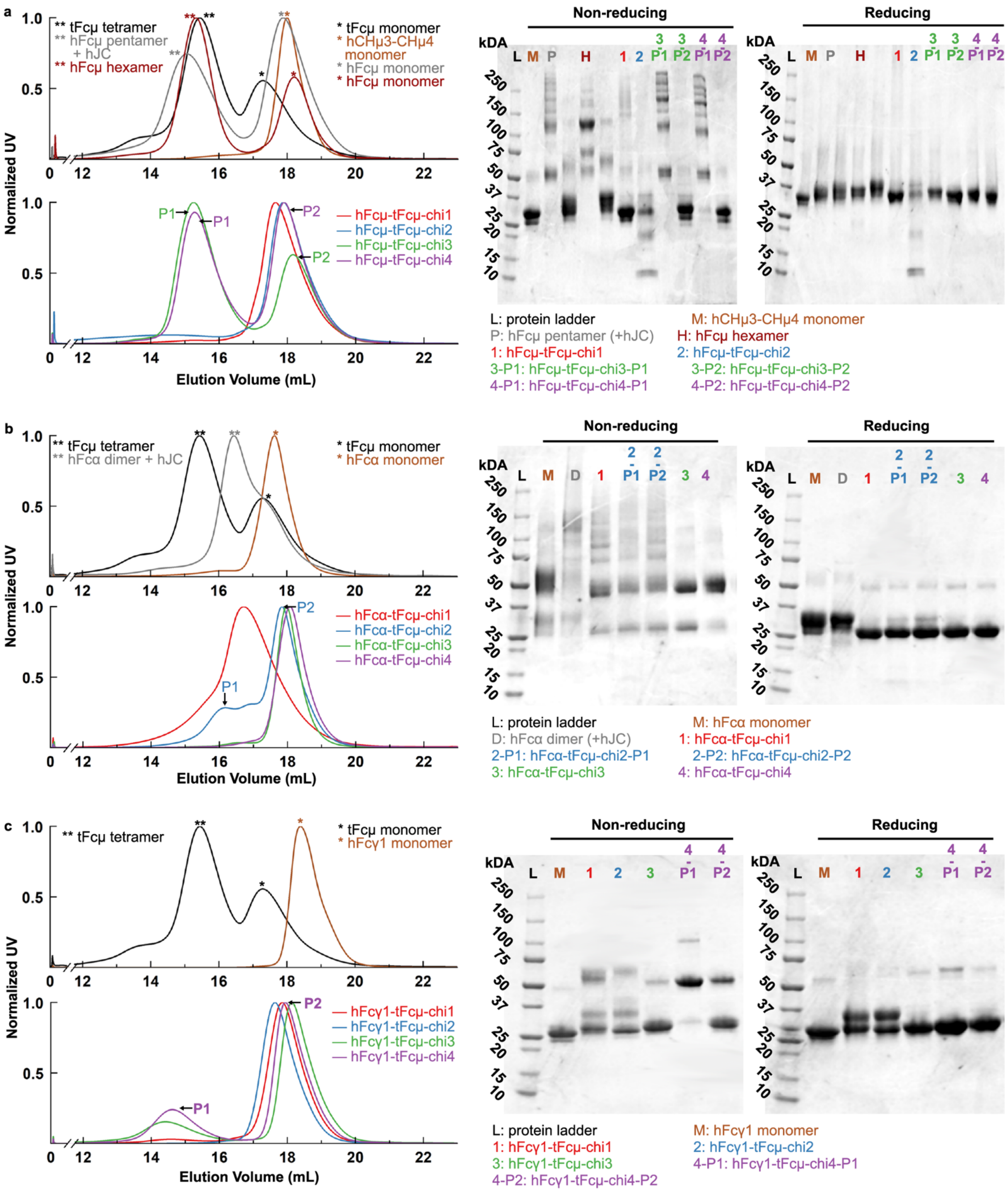
a
** tFcμ tetramer
** hFcμ pentamer + hJC
** hFcμ hexamer
* tFcμ monomer
* hCHμ3-CHμ4 monomer
* hFcμ monomer
* hFcμ monomer
Normalized UV
Elution Volume (mL)
P1
P2
— hFcμ-tFcμ-chi1
— hFcμ-tFcμ-chi2
— hFcμ-tFcμ-chi3
— hFcμ-tFcμ-chi4
Non-reducing
Reducing
kDA
L M P H 1 2 3-P1 3-P2 4-P1 4-P2
L: protein ladder
P: hFcμ pentamer (+hJC)
1: hFcμ-tFcμ-chi1
3-P1: hFcμ-tFcμ-chi3-P1
4-P1: hFcμ-tFcμ-chi4-P1
M: hCHμ3-CHμ4 monomer
H: hFcμ hexamer
2: hFcμ-tFcμ-chi2
3-P2: hFcμ-tFcμ-chi3-P2
4-P2: hFcμ-tFcμ-chi4-P2
b
** tFcμ tetramer
** hFcα dimer + hJC
* tFcμ monomer
* hFcα monomer
Normalized UV
Elution Volume (mL)
P1
P2
— hFcα-tFcμ-chi1
— hFcα-tFcμ-chi2
— hFcα-tFcμ-chi3
— hFcα-tFcμ-chi4
Non-reducing
Reducing
kDA
L M D 1 2-P1 2-P2 3 4
L: protein ladder
D: hFcα dimer (+hJC)
2-P1: hFcα-tFcμ-chi2-P1
3: hFcα-tFcμ-chi3
M: hFcα monomer
1: hFcα-tFcμ-chi1
2-P2: hFcα-tFcμ-chi2-P2
4: hFcα-tFcμ-chi4
c
** tFcμ tetramer
* tFcμ monomer
* hFcγ1 monomer
Normalized UV
Elution Volume (mL)
P1
P2
— hFcγ1-tFcμ-chi1
— hFcγ1-tFcμ-chi2
— hFcγ1-tFcμ-chi3
— hFcγ1-tFcμ-chi4
Non-reducing
Reducing
kDA
L M 1 2 3 4-P1 4-P2
L: protein ladder
1: hFcγ1-tFcμ-chi1
3: hFcγ1-tFcμ-chi3
4-P2: hFcγ1-tFcμ-chi4-P2
M: hFcγ1 monomer
2: hFcγ1-tFcμ-chi2
4-P1: hFcγ1-tFcμ-chi4-P1

Supplementary Fig. 6 | Purification of hFc-tFcμ chimeric Fcs a-b The **left** graph in each panel includes SEC chromatographs of hFc-tFcμ chimeras and relevant controls obtained during purification and prior to SEC-MALS experiments. For controls, peaks containing presumptive monomers are indicated with a single asterisk and peaks containing higher order polymers are indicated with two asterisks. For chimeras with more than one peak, "P1" designates higher order polymers and "P2" designates presumptive monomers. For each sample, elution fractions located at the center of each peak were and run on the SDS-PAGE gels under non-reducing and reducing condition, the results of which are shown in the **right** graph in each panel. **a** hFcμ-tFcμ chimeras and relevant controls. **b** hFcα-tFcμ chimeras and relevant controls. **c** hFcγ1-tFcμ chimeras and relevant controls. The two bands observed for reduced samples hFcγ1-tFcμ-chi1 and hFcγ1-tFcμ-chi2 are attributed to heterogeneous glycosylation. Source data are provided as a Source Data file. Protein preparation, SDS-PAGE and SEC analysis has been repeated at least twice for each sample.

Supplementary Table 1. Cryo-EM data collection and refinement statistics associated with tFcμ

**Accession codes**		**Model**	
EM Databank	EMD-40054	Refinement software	PHENIX[46,47] and Coot[45]
Protein Databank	8GHZ	Validation software	PHENIX[46,47]
		Chains	16
**Data Collection**		Total atoms	28,463
Magnification	105,000	Non-hydrogen atoms	14,390
Pixel size (Å)	0.427	Residues	1,768
Frames per movie	40	Bond RMSD	
Movies selected	3,618	Length (Å)	0.004
Defocus range (μm)	0.5-5	Angle (°)	1.036
Dosage (e/Å^2)	60	MolProbity score	1.95
Initial particles	1,928,186	Clash score	6.95
Final particles	610,190	Ramachandran plot (%)	
		Favored	91.89
**Resolution estimate (unmasked data)**		Allowed	8.11
Software	PHENIX[46,47]	Outliers	0
FSC (model)=0.143 (Å)	2.79	Rotamer outliers (%)	1.28
FSC (half map 1,2)=0.143 (Å)	2.86	Cβ outliers (%)	0
		Peptide plane (%)	
**Density and Fit**		Cis proline	0
Model/Data CC		Twisted proline	0
Mask	0.79	CaBLAM outliers (%)	3.57
Box	0.78	EMRinger score	3.09
Peaks	0.65		
Volume	0.78		

Supplementary Table 2. tFcμ and hFcμ PISA calculations

Chain 1	Chain 2	Interface Area (Å^2)	Interface ∆G (kcal/mol)	Interface ∆G P - value
Interfaces between teleost IgM CH4 domain				
**A**	B	917.5	-7.54	0.36
**C**	D	928.5	-7.19	0.31
**E**	F	904.3	-9.60	0.19
**G**	H	914.9	-8.60	0.31
**B**	C	856.8	-2.73	0.61
**D**	E	729.4	-3.60	0.43
**F**	G	812.2	-3.61	0.55
Interfaces between human IgM CH4 domain				
**A**	B	1216.5	-18.92	0.06
**C**	D	1167.7	-17.20	0.11
**E**	F	1159.7	-20.98	0.04
**G**	H	1194.1	-14.29	0.22
**K**	L	1182.8	-17.45	0.12
**B**	C	447.5	-0.24	0.70
**D**	E	400.9	-0.75	0.66
**F**	G	372.7	-2.54	0.50
**H**	K	382.2	-2.40	0.55

Supplementary Table 3. Diameter measurements for the solvent accessible hole at the center of tFcμ structure

Atom 1	Atom 2	Distance
**Thr428$^{Fc\mu 2D}$/CB**	Asn433$^{Fc\mu 3E}$/HB2	12.07Å
**Thr428$^{Fc\mu 3E}$/CB**	Asn433$^{Fc\mu 2D}$/HB3	12.89Å
**Asn430$^{Fc\mu 2D}$/CA**	Pro432$^{Fc\mu 3E}$/C	12.36Å
**Ser429$^{Fc\mu 2D}$/CB**	Asn433$^{Fc\mu 4G}$/HB2	12.11Å
**Thr428$^{Fc\mu 3E}$/O**	Asn433$^{Fc\mu 2D}$/HB3	11.47Å
**Asn430$^{Fc\mu 3E}$/CA**	Pro432$^{Fc\mu 2D}$/C	13.52Å
**Asn430$^{Fc\mu 3E}$/H**	Asn433$^{Fc\mu 2D}$/CB	11.99Å
**Asn433$^{Fc\mu 3E}$/N**	Ser429$^{Fc\mu 2D}$/O	11.15Å
**Asn433$^{Fc\mu 3E}$/CB**	Asn430$^{Fc\mu 2D}$/H	12.40Å
		**Avg.: 12.2Å**

Supplementary Table 4. MALS data summary

Sample description	Protein MW calculated from sequence (kDa)	Average protein MW from MALS data (Da)	TOTAL number of PNGS* (prediction potential**>0.7)	TOTAL number of PNGS (prediction potential>0.5)	Number of PNGS on each HC: (prediction potential>0.7)	Number of PNGS on each HC: (prediction potential>0.5)
**tFcμ tetramer**	215.7	2.766×10^5 (±1.417%)	0	24	1	3
**hCHμ3-CHμ4**	49.6	4.338×10^4 (±0.543%)	0	4	0	2
**hFcμ pentamer + hJC**	285.1	2.959×10^5 (±0.106%)	11	31	1	3
**hFcμ hexamer**	340.4	3.156×10^5 (±0.073%)	12	36	1	3
**hFcμ-tFcμ-chi1**	54.0	3.859×10^4 (±0.499%)	0	6	0	3
**hFcμ-tFcμ-chi2**	54.5	3.310×10^4 (±3.953%)	0	6	0	3
**hFcμ-tFcμ-chi3-P1**	267.5	2.708×10^5 (±0.077%)	0	24	0	3
**hFcμ-tFcμ-chi3-P2**	53.5	3.748×10^4 (±0.387%)	0	6	0	3
**hFcμ-tFcμ-chi4-P1**	214.3	2.725×10^5 (±0.076%)	0	24	0	3
**hFcμ-tFcμ-chi4-P2**	53.6	4.283×10^4 (±0.388%)	0	6	0	3
**hFcα dimer +hJC**	67.5	1.377×10^5 (±0.180%)	9	9	1	2
**hFcα monomer**	25.9	6.117×10^4 (±1.187%)	4	4	1	2
**hFcα-tFcμ-chi1**	52.7	1.054×10^5 (±1.097%)	4	8	1	2
**hFcα-tFcμ-chi2-P1**	210.4	2.161×10^5 (±1.352%)	8	16	1	2
**hFcα-tFcμ-chi2-P2**	52.6	6.119×10^4 (±8.826%)	2	4	1	2
**hFcα-tFcμ-chi3**	52.1	4.519×10^4 (±16.598%)	2	4	1	2
**hFcα-tFcμ-chi4**	52.1	4.888×10^4 (±2.909%)	2	4	1	2
**hFcγ1**	49.6	4.723×10^4 (±0.996%)	2	2	1	1
**hFcγ1-tFcμ-chi1**	54.0	5.217×10^4 (±3.975%)	2	4	1	2
**hFcγ1-tFcμ-chi2**	54.5	5.598×10^4 (±5.051%)	2	4	1	2
**hFcγ1-tFcμ-chi3**	53.5	5.481×10^4 (±0.419%)	2	4	1	2
**hFcγ1-tFcμ-chi4-P1**	106.7	4.837×10^5 (±0.091%)	16	32	1	2
**hFcγ1-tFcμ-chi4-P2**	53.4	4.917×10^4 (±0.381%)	2	4	1	2

**PNGS stands for "potential N-linked glycosylation site".*
***Prediction potential is calculated using NetNGlyc 1.0 developed by DTU Health Tech[52].*

Supplementary Table 5. Amino acid sequences of hFc-tFcμ-chi constructs.

CONSTRUCT NAME	AMINO ACID SEQUENCE
**hFcμ-tFcμ-chi1**	HHHHHHSGTAIRVFAIPPSFASIFLTKSTKLTCLVTDLTTYDSVTISWTRQNGEAVKTHTNISE<br>SHPNATFSAVGEASICEDDWNSGERFTCTVTHTDLPSPLKQTISRPKGVALHRPDVYLLPPARE<br>QLNLRESATITCLVTGFSPADVFVQWMQRGQPLSPEKYVTSAPMPEPQAPGRYFAHSILTVSEE<br>EWNTGETYTCVVAHESMIKSTNIIMRTIDRTSNQPNLVNLSLNVPQRCMAQ
**hFcμ-tFcμ-chi2**	HHHHHHSGTAIRVFAIPPSFASIFLTKSTKLTCLVTDLTTYDSVTISWTRQNGEAVKTHTNISE<br>SHPNATFSAVGEASICEDDWNSGERFTCTVTHTDLPSPLKQTISRPKGVALHRPDVYLLPPARE<br>QLNLRESATITCLVTGFSPADVFVQWMQRGQPLSPEKYVTSAPMPEPQAPGRYFAHSILTVSEE<br>EWNTGETYTCVVAHEALPNRKSTNIIMRTIDRTSNQPNLVNLSLNVPQRCMAQ
**hFcμ-tFcμ-chi3**	HHHHHHSGTAIRVFAIPPSFASIFLTKSTKLTCLVTDLTTYDSVTISWTRQNGEAVKTHTNISE<br>SHPNATFSAVGEASICEDDWNSGERFTCTVTHTDLPSPLKQTISRPKGVALHRPDVYLLPPARE<br>QLNLRESATITCLVTGFSPADVFVQWMQRGQPLSPEKYVTSAPMPEPQAPGRYFAHSILTVSEE<br>EWNTGETYTCVVAHEALPNRVTERTVDRTSNQPNLVNLSLNVPQRCMAQ
**hFcμ-tFcμ-chi4**	HHHHHHSGTAIRVFAIPPSFASIFLTKSTKLTCLVTDLTTYDSVTISWTRQNGEAVKTHTNISE<br>SHPNATFSAVGEASICEDDWNSGERFTCTVTHTDLPSPLKQTISRPKGVALHRPDVYLLPPARE<br>QLNLRESATITCLVTGFSPADVFVQWMQRGQPLSPEKYVTSAPMPEPQAPGRYFAHSILTVSEE<br>EWNTGETYTCVVAHEALPNRIIMRTIDRTSNQPNLVNLSLNVPQRCMAQ
**hFcα-tFcμ-chi1**	HHHHHHSGCHPRLSLHRPALEDLLLGSEANLTCTLTGLRDASGVTFTWTPSSGKSAVQGPPERD<br>LCGCYSVSSVLPGCAEPWNHGKTFTCTAAYPESKTPLTATLSKSGNTFRPEVHLLPPPSEELAL<br>NELVTLTCLARGFSPKDVLVRWLQGSQELPREKYLTWASRQEPSQGTTTFAVTSILRVAAEDWK<br>KGDTFSCMVGHEALPLAFTNIIMRTIDRTSNQPNLVNLSLNVPQRCMAQ
**hFcα-tFcμ-chi2**	HHHHHHSGCHPRLSLHRPALEDLLLGSEANLTCTLTGLRDASGVTFTWTPSSGKSAVQGPPERD<br>LCGCYSVSSVLPGCAEPWNHGKTFTCTAAYPESKTPLTATLSKSGNTFRPEVHLLPPPSEELAL<br>NELVTLTCLARGFSPKDVLVRWLQGSQELPREKYLTWASRQEPSQGTTTFAVTSILRVAAEDWK<br>KGDTFSCMVGHEALPLAFTNIIMRTIDRTSNQPNLVNLSLNVPQRCMAQ
**hFcα-tFcμ-chi3**	HHHHHHSGCHPRLSLHRPALEDLLLGSEANLTCTLTGLRDASGVTFTWTPSSGKSAVQGPPERD<br>LCGCYSVSSVLPGCAEPWNHGKTFTCTAAYPESKTPLTATLSKSGNTFRPEVHLLPPPSEELAL<br>NELVTLTCLARGFSPKDVLVRWLQGSQELPREKYLTWASRQEPSQGTTTFAVTSILRVAAEDWK<br>KGDTFSCMVGHEALPLAFTQKTIDRTSNQPNLVNLSLNVPQRCMAQ
**hFcα-tFcμ-chi4**	HHHHHHSGCHPRLSLHRPALEDLLLGSEANLTCTLTGLRDASGVTFTWTPSSGKSAVQGPPERD<br>LCGCYSVSSVLPGCAEPWNHGKTFTCTAAYPESKTPLTATLSKSGNTFRPEVHLLPPPSEELAL<br>NELVTLTCLARGFSPKDVLVRWLQGSQELPREKYLTWASRQEPSQGTTTFAVTSILRVAAEDWK<br>KGDTFSCMVGHEALPLAFIMRTIDRTSNQPNLVNLSLNVPQRCMAQ
**hFcγ1-tFcμ-chi1**	HHHHHHSGPSVFLFPPKPKDTLMISRTPEVTCVVVDVSHEDPEVKFNWYVDGVEVHNAKTKPRE<br>EQYNSTYRVVSVLTVLHQDWLNGKEYKCKVSNKALPAPIEKTISKAKGQPREPQVYTLPPSRDE<br>LTKNQVSLTCLVKGFYPSDIAVEWESNGQPENNYKTTPPVLDSDGSFFLYSKLTVDKSRWQQGN<br>VFSCSVMHESMIKSTNIIMRTIDRTSNQPNLVNLSLNVPQRCMAQ
**hFcγ1-tFcμ-chi2**	HHHHHHSGPSVFLFPPKPKDTLMISRTPEVTCVVVDVSHEDPEVKFNWYVDGVEVHNAKTKPRE<br>EQYNSTYRVVSVLTVLHQDWLNGKEYKCKVSNKALPAPIEKTISKAKGQPREPQVYTLPPSRDE<br>LTKNQVSLTCLVKGFYPSDIAVEWESNGQPENNYKTTPPVLDSDGSFFLYSKLTVDKSRWQQGN<br>VFSCSVMHEALHNHKSTNIIMRTIDRTSNQPNLVNLSLNVPQRCMAQ
**hFcγ1-tFcμ-chi3**	HHHHHHSGPSVFLFPPKPKDTLMISRTPEVTCVVVDVSHEDPEVKFNWYVDGVEVHNAKTKPRE<br>EQYNSTYRVVSVLTVLHQDWLNGKEYKCKVSNKALPAPIEKTISKAKGQPREPQVYTLPPSRDE<br>LTKNQVSLTCLVKGFYPSDIAVEWESNGQPENNYKTTPPVLDSDGSFFLYSKLTVDKSRWQQGN<br>VFSCSVMHEALHNHYTQKSLSRTSNQPNLVNLSLNVPQRCMAQ
**hFcγ1-tFcμ-chi4**	HHHHHHSGPSVFLFPPKPKDTLMISRTPEVTCVVVDVSHEDPEVKFNWYVDGVEVHNAKTKPRE<br>EQYNSTYRVVSVLTVLHQDWLNGKEYKCKVSNKALPAPIEKTISKAKGQPREPQVYTLPPSRDE<br>LTKNQVSLTCLVKGFYPSDIAVEWESNGQPENNYKTTPPVLDSDGSFFLYSKLTVDKSRWQQGN<br>VFSCSVMHEALHNHIMRTIDRTSNQPNLVNLSLNVPQRCMAQ

Gray highlight, hexa-histidine tag and Ser-Gly linker. *Yellow highlight*, human Fc sequence. *Blue highlight*, teleost Fcμ sequence.

Original gel image

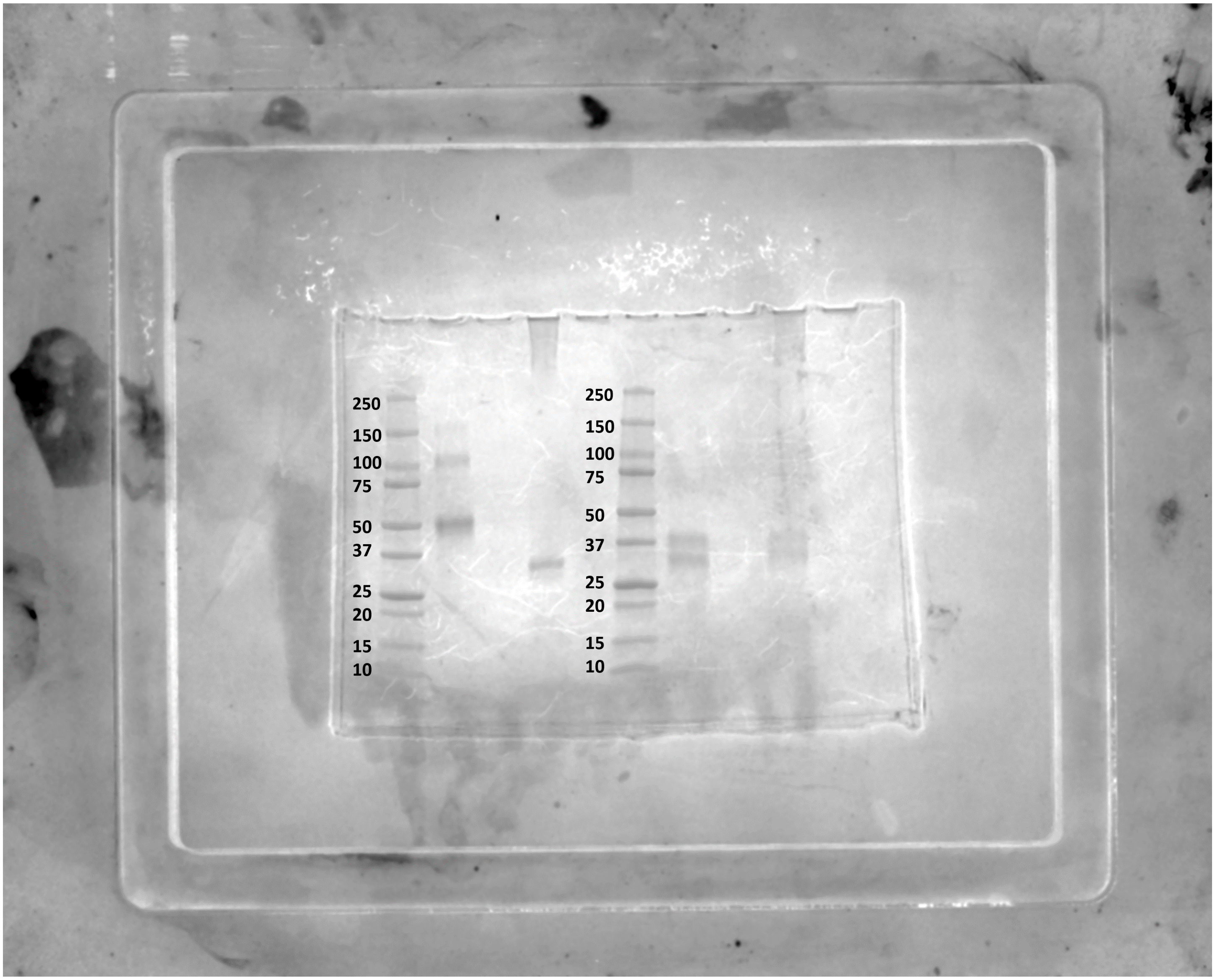

**This gel is shown in Supplementary Fig. 1b and Supplementary Fig. 4g. Molecular weight standards are numbered (kilodaltons).*
