# Supplementary material for: The structure of the teleost Immunoglobulin M core provides insights on polymeric antibody evolution, assembly, and function
Source: Nat Commun. 2023 Nov 21;14:7583. doi: 10.1038/s41467-023-43240-z (PMC10663602; doi:10.1038/s41467-023-43240-z)
Supplement: Supplementary file 3 — Reporting Summary [file 41467_2023_43240_MOESM3_ESM.pdf]

nature portfolio

Corresponding author(s): Beth M. Stadtmueller

Last updated by author(s): Oct 15, 2023

Reporting Summary

Nature Portfolio wishes to improve the reproducibility of the work that we publish. This form provides structure for consistency and transparency in reporting. For further information on Nature Portfolio policies, see our Editorial Policies and the Editorial Policy Checklist.

Statistics

For all statistical analyses, confirm that the following items are present in the figure legend, table legend, main text, or Methods section.

n/a	Confirmed	
x		The exact sample size (n) for each experimental group/condition, given as a discrete number and unit of measurement
	x	A statement on whether measurements were taken from distinct samples or whether the same sample was measured repeatedly
x		The statistical test(s) used AND whether they are one- or two-sided<br>*Only common tests should be described solely by name; describe more complex techniques in the Methods section.*
x		A description of all covariates tested
x		A description of any assumptions or corrections, such as tests of normality and adjustment for multiple comparisons
x		A full description of the statistical parameters including central tendency (e.g. means) or other basic estimates (e.g. regression coefficient) AND variation (e.g. standard deviation) or associated estimates of uncertainty (e.g. confidence intervals)
x		For null hypothesis testing, the test statistic (e.g. F, t, r) with confidence intervals, effect sizes, degrees of freedom and P value noted<br>*Give P values as exact values whenever suitable.*
x		For Bayesian analysis, information on the choice of priors and Markov chain Monte Carlo settings
x		For hierarchical and complex designs, identification of the appropriate level for tests and full reporting of outcomes
x		Estimates of effect sizes (e.g. Cohen's d, Pearson's r), indicating how they were calculated

Our web collection on statistics for biologists contains articles on many of the points above.

Software and code

Policy information about availability of computer code

Data collection	SerialEM
Data analysis	cryoSPARC, RELION, SWISS-MODEL, UCSF Chimera, Coot Molecular Graphics Package, Phenix, PyMOL, PISA (Protein interfaces, surfaces, and assemblies), ASTRA, Origin

For manuscripts utilizing custom algorithms or software that are central to the research but not yet described in published literature, software must be made available to editors and reviewers. We strongly encourage code deposition in a community repository (e.g. GitHub). See the Nature Portfolio guidelines for submitting code & software for further information.

Data

Policy information about availability of data

All manuscripts must include a data availability statement. This statement should provide the following information, where applicable:

- Accession codes, unique identifiers, or web links for publicly available datasets
- A description of any restrictions on data availability
- For clinical datasets or third party data, please ensure that the statement adheres to our policy

The tFcμ tetramer Cryo-EM data generated in this study have been deposited in the EM databank (www.ebi.ac.uk/emdb) with the accession code EMD-40054. The refined coordinates generated in this study have been deposited in the Protein Data Bank (www.rcsb.org) with accession code 8GHZ. The protein structure data used in this study are available in the Protein Data Bank (www.rcsb.org) under PDB ID: 6KXS 6KXS24 (human hSIgM-Fc core), 6UE723 (human hSIgA-Fc core), 1MCO43 (human IgG1-Fc). Source data are provided with this paper. The protein sequences used for sequence alignment are available in NCBI database (https://

www.ncbi.nlm.nih.gov/) under these accession codes: Oncorhynchus mykiss (trout) IgM secretory form (AAW66974.1), Salmo Salar (salmon) IgM (AAB24064.1), Xenopus laevis (frog) IgM (AAA49774.1), Trachemys scripta elegans (turtle) IgM (AFR90255.1), Gallus gallus (chicken) IgM (P01875), Mus musculus (mouse) IgM (P01872), and Homo sapiens (human) IgM (P01871).

Human research participants

Policy information about studies involving human research participants and Sex and Gender in Research.

Reporting on sex and gender	*Use the terms sex (biological attribute) and gender (shaped by social and cultural circumstances) carefully in order to avoid confusing both terms. Indicate if findings apply to only one sex or gender; describe whether sex and gender were considered in study design whether sex and/or gender was determined based on self-reporting or assigned and methods used. Provide in the source data disaggregated sex and gender data where this information has been collected, and consent has been obtained for sharing of individual-level data; provide overall numbers in this Reporting Summary. Please state if this information has not been collected. Report sex- and gender-based analyses where performed, justify reasons for lack of sex- and gender-based analysis.*
Population characteristics	*Describe the covariate-relevant population characteristics of the human research participants (e.g. age, genotypic information, past and current diagnosis and treatment categories). If you filled out the behavioural & social sciences study design questions and have nothing to add here, write "See above."*
Recruitment	*Describe how participants were recruited. Outline any potential self-selection bias or other biases that may be present and how these are likely to impact results.*
Ethics oversight	*Identify the organization(s) that approved the study protocol.*

Note that full information on the approval of the study protocol must also be provided in the manuscript.

Field-specific reporting

Please select the one below that is the best fit for your research. If you are not sure, read the appropriate sections before making your selection.

[x] Life sciences [ ] Behavioural & social sciences [ ] Ecological, evolutionary & environmental sciences

For a reference copy of the document with all sections, see nature.com/documents/nr-reporting-summary-flat.pdf

Life sciences study design

All studies must disclose on these points even when the disclosure is negative.

Sample size	For cryoEM studies, 3618 micrographs (movies) were collected and XXXK particles were used for 3D reconstruction.
Data exclusions	Cryo-EM micrographs with thick ice and low CTF-estimates were excluded in the data processing step as it is standardized procedure.
Replication	For all protein complexes analyzed in this study, expression, purification and size exclusion chromatography (SEC) were conducted at least twice and were found to be in agreement. SEC with in-line multi-angle light scattering (SEC-MALS) experiments on chimeric protein complexes were conducted at least twice. For cryo-EM data sets see "Randomization" section below.
Randomization	The cryoEM dataset was randomly split into two half-sets that were refined independently in cryoSPARC, producing two half-maps. The reported resolution was calculated from the Fourier Shell Correlation between the two half-maps at the 0.143 threshold criteria. This indicates that the resolution is reproducible from this dataset.
Blinding	Blinding was not applicable to this study. For cryoEM data, the splitting of data to half-sets was performed automatically by the processing software (CryoSPARC).

Reporting for specific materials, systems and methods

We require information from authors about some types of materials, experimental systems and methods used in many studies. Here, indicate whether each material, system or method listed is relevant to your study. If you are not sure if a list item applies to your research, read the appropriate section before selecting a response.

Materials & experimental systems

n/a	Involved in the study
☒	☐ Antibodies
☐	☒ Eukaryotic cell lines
☒	☐ Palaeontology and archaeology
☒	☐ Animals and other organisms
☒	☐ Clinical data
☒	☐ Dual use research of concern

Methods

n/a	Involved in the study
☒	☐ ChIP-seq
☒	☐ Flow cytometry
☒	☐ MRI-based neuroimaging

Eukaryotic cell lines

Policy information about cell lines and Sex and Gender in Research

Cell line source(s)	Human embryonic kidney (HEK) Expi293-F cell line (Gibco, A14528) is a transformed cell line with a species of origin Homo sapiens (Human), derived from embryonic kidney tissue. The sex of this cell line is female.
Authentication	Expi293F(Gibco, A14528) was purchased commercially (from Thermo Fisher Scientific) with testing reports. No further authentication was performed.
Mycoplasma contamination	Certificate of analysis provided by Thermo Fisher Scientific shows negative result from qPCR assay.
Commonly misidentified lines (See ICLAC register)	No commonly misidentified cell lines were used in this study.
